# “A Role Model Is Someone Who…” A Multi-institutional Study of Clinical Role Models According to Ethnic Minority and Majority Medical Students

**DOI:** 10.1007/s40670-025-02317-8

**Published:** 2025-02-18

**Authors:** Isabella Spaans, Renske de Kleijn, Piet Groot, Gönül Dilaver

**Affiliations:** 1 Educational Center, University Medical Center Utrecht, Heidelberglaan 100, Postbox 85500, 3508 GA Utrecht, The Netherlands; 2 Utrecht University, Utrecht, The Netherlands; 3 University Medical Center Utrecht and Utrecht University, Utrecht, The Netherlands

**Keywords:** Clinical role models, URiM, Diversity, Workplace learning, Role modeling, Inclusive education

## Abstract

**Purpose:**

It is a common conception that students who are culturally underrepresented in medicine (URiM) do not have enough representative role models. This study explores the role of ethnicity in medical students’ clinical role model definitions. The authors introduce a conceptual framework that outlines a four-stage process of role modeling: idealization, social comparison, composition, and (behavioral and symbolic) outcomes.

**Method:**

In total, 363 Dutch medical students completed the statement “A role model is someone who…” Answers were coded based on the conceptual framework. Students also indicated if and how many role models they have (composition) and rated the ethnic similarity to their role model. URiM (*N* = 62) and non-URiM students (*N* = 301) were compared using *χ*^2^- and *t*-tests.

**Results:**

URiM and non-URiM students reported a similar number of role models and described the same stages of role modeling. However, URiM students rated the ethnic similarity to their role models lower than non-URiM peers. Additionally. students with less ethnically similar role models reported symbolic role model outcomes less frequently.

**Conclusions:**

URiM and non-URiM students generally presented a very similar perception of clinical role models. However, URiM students identified less ethnically representative role models compared to non-URiM students, and the symbolic outcomes of role modeling appeared to be sensitive to this ethnic similarity. This discrepancy may limit the full benefits of role modeling for all students who do not have representative role models. To promote equitable learning experiences in medical education, it is recommended that future research on clinical role models continues to address the social context.

## Introduction

### Role Models in Medical Education

Role modeling is an established teaching method in medical education, where medical students learn by observing more experienced physicians. Role models play an important role in students’ socialization, professional identity development, and medical specialty choice [1–4], making them a powerful factor in medical education [5–9].

Considering the importance of clinical role modeling, it makes sense that medical education scholars have investigated clinical role models extensively. Traditionally, these studies emphasize role models’ *behavioral* value, where physicians model behaviors for students to emulate. Central to these studies are the attributes of excellent clinical teachers. These role model attributes are typically inventoried and then categorized into clinical qualities, teaching qualities, and personal qualities, a trichotomy introduced by Wright in 1997 [2, 10–16].

In more recent years, role modeling has regained scholars’ interest, only this time in the context of the increasingly culturally diverse student body. This line of research emphasizes role models’ *symbolic* value, where role models are seen as inspirational and motivational, representing the possible, and helping students permeate medical schools’ hidden curriculum. For ethnic minority students, also referred to as culturally underrepresented in medicine (URiM), clinical role models who share the same ethnic background (*representative role models*) strengthen their enthusiasm and offer guidance and support [17, 18]. They also help recruit and retain URiM students [19–22].

Conversely, the absence of representative role models is associated with a range of negative outcomes. These include a negative effect on URiM students’ matriculation, experiencing “inadequacy, inc ompetency, and self-doubt” and having limited access to relatable experiences [23, 24]. Additionally, URiM students risk being tokenized which means that a small number of underrepresented individuals are included to create an illusion of equality [25]. Summarizing, representative role models fulfill a critical role in URiM students’ study experience and ultimately in combatting the leaky pipeline phenomenon [26].

Between these two lines of research, where one established the importance of clinical role models and the other the particular significance of representative role models, lies a knowledge gap to be bridged. A systematic, empirical analysis of students’ role model definitions is needed to pinpoint if and how students’ definition of role models is shaped by (1) their own ethnicity and (2) how ethnically representative their role models are.

### Conceptual Framework: The Clinical Role Modeling Process

**Fig. 1:**
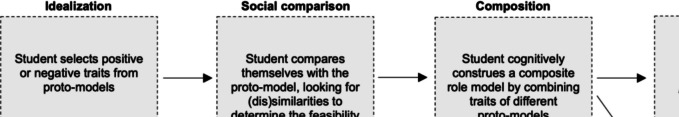
Conceptual framework: schematic representation of medical students’ role modeling process

To compare URiM and non-URiM students’ image of role models and extend our scope beyond clinical role model attributes, we developed a conceptual framework of the broader role modeling process [27] (see Fig. 1). Drawing upon theoretical advancements from various academic disciplines, the framework offers a schematic representation of four stages of role modeling: idealization, social comparison, composition, and behavioral and symbolic outcomes.

### Idealization

The role modeling process starts when students identify positive or negative attributes of the physicians around them. Organizational research on career role models for women refers to this as the *idealization* of *proto-models* [28]. This idealization process builds on the premise of *selective imitation*, where one does not aspire to imitate another person in their entirety, but rather selects specific attributes while ignoring others [29, 30]. Most research on clinical role models focuses on this idealization part of the role modeling process, asking: which attributes do students wish to emulate?

### Social Comparison

The second stage involves social comparison between the student and their role model. In the management theory, social comparison is described as “the processes through which individuals construct their identities, often through comparing and contrasting themselves to others” [31]. The perceived similarity between the student and their role model enables them to assess the feasibility and desirability of following in their role model’s footsteps [32–34]. Although the significance of similarity between diverse medical learners and their role models has previously been stated [35], it is not typically taken into account in studies about clinical role models.

### Composition

The next stage follows from a social constructivist approach to role models and is novel to medical education research. It views a role model not as an actual person, but as the mental representation of attributes from real people (proto-models), selected during the idealization phase [29, 30, 32, 36, 37]. Together, these attributes form a dynamic, ever-evolving mental image of “the perfect physician” that can guide the student through whatever circumstances they are facing. As organizational theorist Zetterquist puts it: “People do not model their behavior on real persons, but on mental constructs they make loosely inspired by actual people” [28]. The cognitive construal of role models largely takes place at a subconscious level.

### Outcomes

Finally, having a role model can result in different and multiple outcomes. First, there are the more tangible *behavioral* outcomes, as role models in medical education are usually viewed. An example of a behavioral outcome is clinical skill acquisition. The behavioral value is also recognized in economics, where it was first distinguished from mentoring and other forms of role modeling [32].

Second, role models carry a more intangible, *symbolic* value, a term coined by organizational researchers. Examples they give of symbolic role model outcomes are increased optimism and trust, commitment, and reduced stereotyping [33]. Psychologists also recognize role models as more symbolic “representations of the possible” and as sources of inspiration [34]. In humanities and philosophy, we see a less dichotomous, more gradual continuum of inspiration as a more sophisticated form of imitation [38]. Symbolic outcomes are particularly important to URiM students because “the outcomes build on the premise that ‘someone like them’ […] can succeed” [37].

## Research Aim

This study aims to investigate how ethnicity, in the form of student migration background and ethnic representativeness of role models, affects how medical students view clinical role models, in the context of Dutch medical universities. The central research question is: *How does students’ role model perception relate to student ethnicity and role model ethnic similarity?* This is important because it will yield the first statistical comparison of empirical data about clinical role model attributes. Moreover, it will allow us to consider essential dimensions such as: do URiM students look for different attributes in role models compared to their non-URiM peers? In what stage of the role modeling process does ethnicity matter?

To address these questions, we developed a conceptual framework, and a survey was employed to collect quantitative data about medical students’ clinical role models. Thus, we aim to take the first step in addressing the role of ethnicity in clinical role modeling. In doing so, we contribute to the ongoing discussions about equitable representation in medical education, not only in The Netherlands or in Europe, but also in global practice.

## Materials and Methods

### Questionnaire Development

To ensure a wide pool of data, we carried out a survey. We piloted our questionnaire with a group of medical students, which resulted in minor adjustments, for example the phrasing of some questions. The questionnaire was in English and was presented in Qualtrics. The Faculty Ethics Assessment Committee Humanities (FEtC-H) reviewed and approved the final survey under number 21–0388, and all respondents signed an informed consent form ahead of participation. We designed four questions, each of which provided information to inform different variables (see Table 1).

**Table 1:**
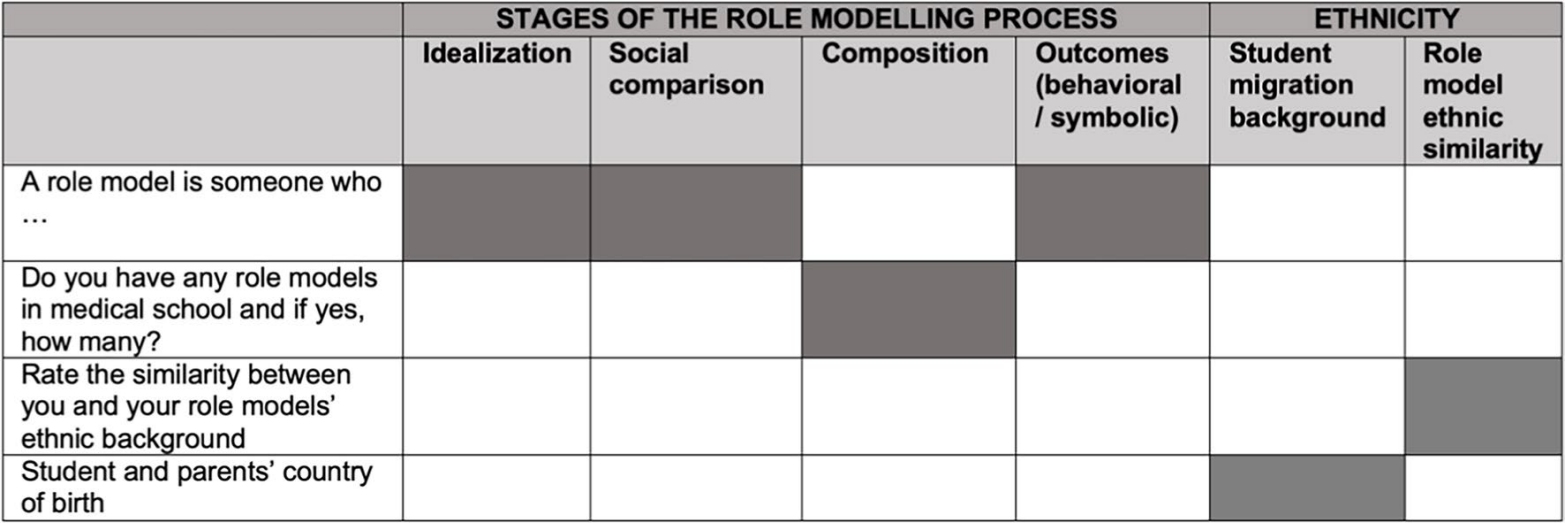
Survey questions and the corresponding informed variables

We started by asking students to complete the statement “A role model is someone who…,” to explore how different medical students view role models. We then asked if the respondents had any role models in medical school and how many, to empirically determine whether URiM students indeed have fewer role models than non-URiM students. The third question was to rate on a 7-point Likert scale the similarity between their and their role model’s ethnic background, ranging from 1 “very dissimilar” to 7 “very similar.” Finally, we inventoried participants’ and their parents’ country of birth to establish their migration background (URiM or non-URiM).

## Participants and Procedure

The population we wanted to study were medical students at all the university medical schools in The Netherlands. We employed a convenience sampling technique where we emailed all eight Dutch medical schools and their student associations, asking them to invite their students to partake in the survey. One medical school granted us permission to email all students individually, including one reminder, and to promote the survey during an online lecture. All the student associations distributed the survey through email or social media channels like Instagram and LinkedIn. The data collection then progressed to snowball sampling, where students forwarded the invitation through WhatsApp. Finally, to secure a sufficient amount of URiM participants, we approached three URiM student associations. The survey closed after 6 weeks.

### Defining URiM

Since our study is conducted in The Netherlands, we followed the definition from Statistics Netherlands to determine our participants’ migration background, acknowledging that underrepresented populations vary by region [39]. Participants had a migration background (and were thus considered URiM) if they were born in a country outside The Netherlands or had at least one parent who was born in a country outside The Netherlands. More precisely, to most accurately represent the URiM students that were the focus of this study, we only included participants with a *non-Western* migration background in the analyses, excluding participants with a Western migration background. [40] Participants were considered non-URiM if they and both of their parents were born in The Netherlands. 

## Qualitative Analysis

To analyze responses to the open-ended query “A role model is someone who…,” we developed a coding scheme that utilized both deductive (theory-based) and inductive (data-driven) approaches.

Initially, we employed deductive codes derived from our conceptual framework. These theoretical elements were later re-labeled to better align with the anticipated practice-oriented nature of students’ responses:

- Idealization was re-labeled as admiration.
- Social comparison was re-labeled as representation.
- Behavioral outcomes were re-labeled as imitation.
- Symbolic outcomes were re-labeled as inspiration.

However, these deductive codes did not fully capture the students’ answers, leaving some data uncoded. To address this, we introduced a round of inductive coding. This process identified additional categories from the data: skilled, example, want to be like, motivation, look up to, background similarity, and goal similarity.

We then integrated both deductive and inductive codes into a final coding scheme with five overarching categories (see Table 2). This final coding scheme covered almost all the data, leaving less than 5% uncoded.

**Table 2:** Overview of the role modeling stages, the corresponding overarching codes, the related theoretical and data subcodes, and the results from two round of intercoder reliability

Role modeling stage	Overarching code	Theory codes	Data codes	Intercoder reliability (*κ*)	
				1st round	2nd round
Idealization	**Clinical Role Model Attributes**	Clinical qualities Teaching qualities Personal qualities	Skilled	0.56	0.73
	**Admiration**	Admiration	Look up to	1.00	0.91
Social comparison	**Representation**	Representation	Background similarity Goal similarity	-	-
Behavioral outcomes	**Imitation**	Imitation	Example Want to be like	0.70	0.86
Symbolic outcomes	**Inspiration**	Inspiration	Motivation Representing the possible	0.84	0.86

### Intercoder Reliability

To ensure intercoder reliability, the first author initially coded half of the data, followed by an independent second investigator coding the same portion. This resulted in moderate agreement (Cohen’s Kappa) for the CRMA code [41]. After refining the coding scheme—particularly by distinguishing between clinical qualities, teaching qualities, and personal qualities—the first author re-coded the entire dataset. When the independent investigator then coded 20% of the data, intercoder reliability improved to good agreement for CRMA, and very good agreement for emulation, inspiration, and admiration. Reliability for representation could not be assessed due to insufficient occurrences.

## Quantitative Analysis

We conducted statistical analyses to compare URiM and non-URiM students’ answers when the frequencies allowed this. First, to compare the frequencies of the role model attributes reported by URiM and non-URiM students, we employed *χ*^2^ tests. Second, to compare the perceived similarity that URiM and non-URiM reported, we employed independent-sample *T*-tests. Third, we analyzed whether ethnic similarity was related to students’ role model definitions by performing independent-sample *T*-tests.

A *p*-value < 0.05 was considered statistically significant and the minimum group size to perform statistical analysis was 10. All analyses were performed using SPSS Statistics 28.0 software (IBM Corporation, Armonk, NY).

## Results

### Study Sample and Descriptive Statistics

Out of the 672 responses to our survey, 363 students completed the statement “A role model is someone who…” and were included in the analysis. Despite purposive sampling, our sample was predominantly non-URiM (301/363, 82.9%), reflecting the underrepresented nature of underrepresented in medicine (URiM) communities. A majority of 54.8% (199/363) of all students had one or more role models in medical school.

Next, we will present the results of the qualitative analysis of students’ role model definitions. Then, we will share how students rated the ethnic representativeness of their role models. Finally, we will relate these findings to ethnicity in the form of students’ migration background and the ethnic representativeness of their role models, to gain insight into differences and similarities between URiM and non-URiM’s role models.

### A Role Model Is Someone Who…

The open-ended statement “A role model is someone who…” elicited a broad range of responses that covered all role modeling stages. Table 3 provides an overview of the role modeling stages and corresponding codes for all students, and for non-URiM and URiM students separately.

**Table 3:** Overview of the main variables categorized per role modeling stage for all students, URiM students and non-URiM students

a	**Total (*n* = 363)**		**Non-URiM (*n* = 301)**			**URiM (*n* = 62)**		
**Role modeling stage**	***n***	**%**	***n***	**%**		***n***	**%**	
**Idealization**								
Clinical role model attributes	113	31.1	91	30.2		22	35.5	
Admiration	87	24.0	74	24.6		13	21.0	
Idolization	5	1.4	4	1.3		1	1.6	
**Social comparison**								
Representation	6	1.7	3	1.0		3	4.8	
**Composition**								
One role model	32	18.5	27		18.4	5		19.2
More than one role model	141	81.5	120		81.6	21		80.8
**Behavioral outcomes**								
Imitation	156	43.0	130	43.2		26	41.9	
**Symbolic outcomes**								
Inspiration	69	19.0	59	19.6		10	16.1	

*N* number of students, whose total percentage may exceed 100% due to simultaneous coding.

### Idealization

The responses to the open-ended statement “A role model is someone who…” most often referred to the idealization stage of role modeling (53.2%, 193/363). This stage involves identifying positive and negative attributes in proto-models. Idealization was coded as clinical role model attributes, admiration and the newly identified idolization. Some students provided attributes specific enough to code with the trichotomy of clinical qualities (e.g., “Behaves well with patients”), teaching qualities (e.g., “Cares about their students”), and personal qualities (e.g., “Has a balanced life”). Other students described role models in more generic ways like admiration (e.g., “A role model is someone I look up to”) or skilled (e.g., “Is good at what they do”). Finally, we identified the new theme idolization, whereby a small subset ascribed unrealistic attributes to their role models, e.g., “a perfect model in [sic] all fronts.”

### Social Comparison

With 1.9% (7/363), social comparison was the least mentioned role modeling stage. Social comparison involves assessing the differences and similarities with one’s role model and consequently the feasibility of following in their footsteps. We operationalized this stage of the role modeling process with the code Representation. One student wrote “Has resemblances to you but is further in their career and achieved some impressive things.”

### Composition

Composition reflects the aggregation of proto-model attributes into a composite role model and was measured by the number of role models students identified. Roughly 18.5% (32/173) of all students named only one role model, but the majority of 81.5% (142/173) had multiple role models. On average, students identified between two and three role models (*M* = 2.75, SD = 1.7).

### Role Modeling Outcomes

Some students also defined a role model by their outcomes, describing what having a role model brings them.

#### Behavioral

Behavioral outcomes are the more tangible results of the role modeling process, like acquiring clinical skills through observation and imitation. We operationalized behavioral outcomes with the code Imitation. With nearly half of all students (172/363, 43%) describing a role model as someone who they wish to emulate, this was the second-most mentioned role modeling stage: for example, “someone I want to be like” or “someone who is an example.”

#### Symbolic

Symbolic outcomes are the less tangible results of the role modeling process, like challenging peoples’ ideas of what is possible and offering hope and inspiration. We operationalized symbolic outcomes with the code Inspiration. 19% (69/363) described a role model as someone who inspires, motivates, or represents the possible, e.g., “Paves the way for others,” “to show what is possible,” and “Make goals seem attainable.”

#### URiM and Non-URiM students’ Role Models

##### Number of Role Models

Despite expectations, URiM students did not identify fewer role models than non-URiM students. More than half of both URiM (32/62, 51.6%) and non-URiM students (167/301, 55.5%) were able to identify role models in medical school (*χ*^2^ = 0.311, *p* = 0.577, *v* = 0.029). An independent-samples *T*-test showed no significant difference in the average number of role models of URiM (*M* = 2.69, SD = 1.51) and non-URiM students (*M* = 2.76, SD = 1.75) (*t* = − 0.172, *p* = 0.864, *d* = − 0.037).

#### Ethnic Representativeness

Although a mere 1.9% (7/363) of all students spontaneously mentioned the representativeness of their role model, nearly all students with role models rated the similarity of their role model’s ethnic background to their own (1 = very dissimilar, 7 = very similar). Independent-samples *T*-tests showed that URiM students rated their role model as less ethnically representative (*N* = 31, *M* = 2.97, SD = 2.03) than non-URiM students (*N* = 165, *M* = 4.61, SD = 1.6) (*t* = − 5.120, *p* = < 0.001, *d* = − 1.002). Only 22.6% (7/31) of URiM students rated their role model as somewhat to very similar, versus 56.4% (93/165) of non-URiM students.

#### Role Modeling Stages

Chi-square tests showed no significant relation between students’ migration background or role model ethnic similarity and the role modeling stages idealization, composition, and behavioral outcomes. Two results were of statistical significance. First, more URiM than non-URiM students named the importance of social comparison between them and their role model. However, this was based only on a small number of occurrences (*n* = 6). Second, the results of the *T*-tests showed a relation with a small to moderate effect size (*t* = − 2.064, *p* = 0.040, *d* = − 0.359) between ethnic similarity and symbolic outcomes. It indicated that students who described role models as inspiring (a symbolic role model outcome) reported higher ethnic similarity to their role model than students who did not describe role models as inspiring (Table 4).

**Table 4:** Role modeling stages in relation to migration background and perceived ethnic similarity

Role modeling stage	Migration background		Role model ethnic similarity	
	***χ***^**2**^**/*t***	***p***	***t***	***p***
Idealization	*χ*^2^ = 0.000	0.992	− 0.400	0.690
Social comparison	-	-	-	-
Composition (number of role models)	t = − 0.172	0.864	–	–
Behavioral outcomes	*χ*^2^ = 0.003	0.856	0.257	0.798
Symbolic outcomes	*χ*^2^ = 0.403	0.526	− 2.064	**0.040**

- not tested due to small group sizes.

– only students with role models rated ethnic similarity.

## Discussion

Our study provided valuable insights into how ethnicity, encompassing both student migration background and the ethnic representativeness of role models, influences medical students’ perceptions of clinical role models.

While it provided empirical validation for the dearth of representative role models for URiM students, it also showed that, contrary to the initial hypothesis, URiM students identified as many clinical role models as non-URiM students (possibly also with mentors in mind). The study highlighted differences in the reported role modeling stages between URiM and non-URiM students, particularly in social comparison and symbolic outcomes, though these differences were based on a small sample size. Lastly, this study serves as a benchmark for future research into clinical role models.

## Challenges of Reflecting on Social Comparison and Symbolic Role Modeling Outcomes

Although social comparison is a fundamental component of role modeling, it was interestingly the least mentioned aspect in students’ definitions of role models. Students rarely described their role models as someone who looks like them or shares the same ethnic background. Similarly, students only sporadically described role models’ symbolic value in terms other than “inspirational” or “motivational.”

For URiM students, we anticipated a reluctancy to report social comparison with their role model or mention symbolic outcomes such as “reduced stereotyping.” This reluctance likely stems from a desire to distance themselves from the stigma associated with their minority status [37]. Identifying with a minority senior role model can be unappealing if they are also still a minority [42].

However, non-URiM students also rarely mentioned social comparison. It seems that both URiM and non-URiM students might overlook this part of the role modeling process because social comparison often occurs subconsciously [37, 43]. Indeed, when we *explicitly* asked students to rate their role models’ similarity, they were able to make social comparisons. Additionally, reflecting on role models’ symbolic value can be challenging, as the significance of an action is often recognized only in hindsight [44].

## Theoretical Contribution of the Conceptual Framework

Our conceptual framework through its corresponding coding scheme demonstrated its added value in two ways. First, it enabled us to code 65% more role model definitions than we could using only the traditional trichotomy of clinical role model attributes [10, 16], which covered just 24% of the data. Consequently, this broader approach to role modeling left only a small part of the data uncoded.

Second, the framework effectively served our research purpose by revealing that the differences between URiM and non-URiM students did not lie in the idealization stage (which encompasses the clinical role modeling attributes), but in the symbolical outcomes. Without this this study’s novel approach, this difference would have remained unobserved. Therefore, we advocate for a broader understanding of role modeling, extending beyond role model attributes, to more accurately capture clinical role modeling from a students’ perspective.

## Finetuning the Conceptual Framework: Idealization, Admiration, and Idolization

Some students used larger-than-life descriptions to define their role models. It is important to distinguish these from admiration, as the unrealistic nature of such accounts can be counterproductive. When a role model represents something unattainable, students may become demoralized, doubting their ability to follow in their role models’ footsteps. [45] Sociologists Bucher and Stelling have referred to these distant role models as charismatic role models. [46] In social psychology this “unrealistic aggrandizement” of a person is called idealization.

In our conceptual framework, we adapted idealization from management theory where it describes the identification of both positive and negative attributes. To differentiate, we refer to unrealistic aggrandizement as *idolization*. Thus idealization (selecting positive or negative attributes) serves as the overarching category, encompassing the subcategories of admiration (identifying positive attributes) and idolization (unrealistic aggrandizement of a person).

## Regional Variations in URiM Populations

The concept of underrepresented in medicine (URiM) varies significantly across different countries. In The Netherlands, URiM is primarily defined by migration background, specifically focusing on individuals with a non-Western migration background. In this context, we define non-Western migration backgrounds as individuals with origins from countries in Africa, Latin America, and Asia (excluding Indonesia and Japan), as well as from Turkey. The rationale for excluding Indonesia and Japan is based on their socio-economic and socio-cultural position, which aligns them more closely with Western migration backgrounds [47]. However, the term URiM holds different meanings in other countries. For instance, in the USA, URiM typically refers to racial and ethnic groups that are underrepresented in the medical profession relative to their proportion in the general population, such as Black, Hispanic, and Native American populations.

This difference in the definition of URiM highlights the need for caution when generalizing findings across different national contexts. While our study focuses on URiM as defined in The Netherlands, the findings may offer insights that can be explored within broader frameworks in other countries, but it is essential to consider the specific socio-political and historical contexts that shape how URiM populations are identified and addressed in each region.

## Strengths, Limitations, and Suggestions for Future Research

Our open-ended statement about what a role model is yielded a wide range of role model definitions, extending beyond clinical role model attributes. This approach provided novel insights into the differences between URiM and non-URiM students. It also revealed that students might benefit from more guidance when reflecting on the role modeling process. Therefore, we suggest using pre-defined answer options in future quantitative studies to explore social comparison between students and their role models, as well as symbolic role model outcomes.

Additionally, the arrows in the proposed conceptual framework suggest a linearity where in reality, role modeling is a highly complex, non-linear, subjective, dynamic, partly subconscious, iterative process. Future research may investigate the accuracy of these arrows in representing the order of events.

Including the newly identified concept of idolization as a deductive code in future research will help explore how exaggerated appraisal of role models may prevent students from benefitting from role modeling.

Role modeling holds particular significance in medical school as a fundamental and well-recognized teaching method. Future studies should examine the applicability of the conceptual framework in educational settings beyond medical school, as well as in other countries beyond the Netherlands.

A limitation of this study is the relatively low response rate among URiM students, which may affect the generalizability of the findings within this subgroup. This low participation is a concern, as it limits the extent to which we can draw definitive conclusions about the experiences and perspectives of URiM students in relation to role models. To improve participation, we did implement targeted outreach efforts aimed at URiM students, such as personalized recruitment messages and collaboration with student organizations that focus on this group. Future research may consider offering incentives, such as small stipends, to increase motivation to participate and ensure a more representative sample. However, it is important to acknowledge that the small portion of URiM students in the sample is an inevitable byproduct of their underrepresentation within the medical student population.

## Conclusions

This study is the first to empirically compare clinical role models among ethnic minority and majority medical students and the first to consider elements of the role modeling process beyond role model attributes. We aimed to answer the research question: *How does students’ role model perception relate to student ethnicity and the ethnic similarity of their role models?*

Our comparison of URiM and non-URiM students’ role model definitions revealed that URiM students do not view role models inherently different from non-URiM students. Instead, their perception of role models appears to be related to the representativeness of these role models: URiM students rated the ethnic similarity to their role models lower than non-URiM students, and the symbolic outcomes of role modeling appeared to be sensitive to this ethnic similarity, possibly limiting the full benefits of role modeling for all students who lack representative role models. Therefore, addressing the broader aspects of role modeling, including ethnicity and the symbolic impact, is recommended to foster equitable learning opportunities in medical education.

## Figures and Tables

**Fig. 2:**
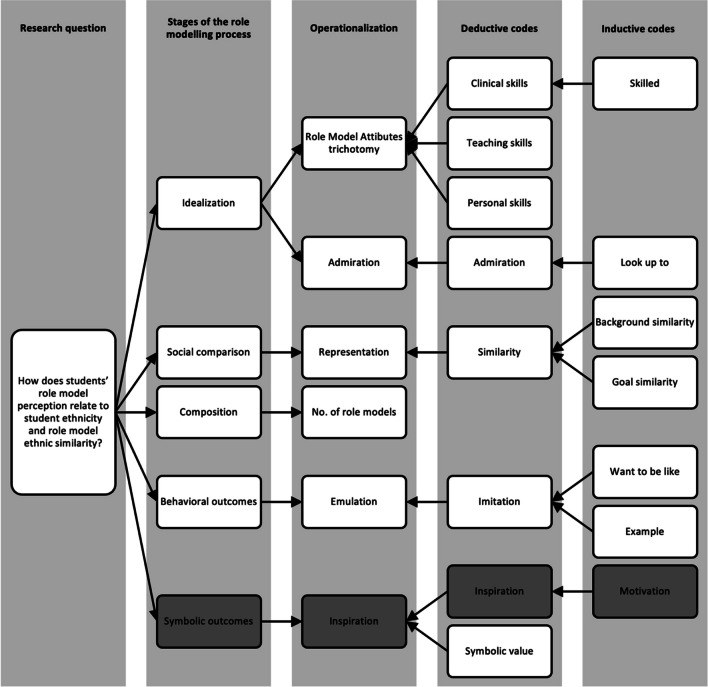
Visual representation of research design and ethnicity-related elements of the role modelinsg process

## Appendix 1

Table 5

**Table 5:** List of variables

Variables	Questions	Values
1. Role model questions		
1a. Role model definition	Complete the following sentence: A role model is someone who ...	• Imitation • Inspiration • Admiration • Representation
1b. Role models in medical school	Do you have role models in medical school? If yes, please enter how many role models you have.	• Yes, [number 1-100] • No
1a. Similarity ethnic backgrounds	My role model(s) and I have similar ethnic backgrounds	• Very dissimilar • Dissimilar • Somewhat dissimilar • Neutral • Somewhat similar • Similar • Very similar
2. Demographic data		
2a. Gender	What gender do you identify as most?	• Male • Female • Non-binary / third gender • Prefer not to say
2b. Student’s country of birth	Were you born in the Netherlands? If not, please enter your country of birth.	• Yes • No, …
2c. Parents’ country of birth	Do you have one parent or more who is born outside of the Netherlands? If yes, please enter the country of birth of your parents.	• Yes, one parent: … • Yes, both parents: … • No
2d. Location educational institute	What is the location of your educational institute?	• Utrecht • Amsterdam • Groningen • Leiden • Maastricht • Rotterdam • Nijmegen • Other: …

## Appendix 2

Figure 2

## Appendix 3

Table 6

**Table 6:** Sample description study year and geographic origin

	***n***	**%**
Study year *1* *2* *3* *4* *5* *6* *7* *8* total	60 58 49 98 61 24 11 2 363	16.5 16.0 13.5 27.0 16.8 6.6 3.0 .6 100
Geographic origin *Netherlands* *Africa* *Asia* *South America* *Mixed* total	301 7 28 15 12 363	82.9 1.9 7.7 4.1 3.3 100
